# The complete mitochondrial genome of the Jackson’s leaf butterfly *Mallika jacksoni* (Insecta: Lepidoptera: Nymphalidae)

**DOI:** 10.1080/23802359.2020.1814885

**Published:** 2020-09-03

**Authors:** Mackenzie R. Alexiuk, Jeffrey M. Marcus, Melanie M. L. Lalonde

**Affiliations:** Department of Biological Sciences, University of Manitoba, Winnipeg, Canada

**Keywords:** Illumina sequencing, mitogenomics, Lepidoptera, Nymphalidae, Kallimini

## Abstract

The Jackson’s leaf butterfly *Mallika jacksoni* (Sharpe 1896), is a leaf-mimicking species from tropical East Africa. Genome skimming by Illumina sequencing permitted the assembly of the complete circular *M. jacksoni* 15,183 bp mitogenome. It consists of 79.4% AT nucleotides, 22 tRNAs, 13 protein-coding genes, 2 rRNAs, and a control region in the typical butterfly gene order. *Mallika jacksoni COX1* has a CGA start codon while *ATP6*, *COX1*, *COX2*, *ND3*, *ND4*, and *ND5* exhibit partial stop codons completed by 3′-A residues added to the mRNA. Phylogenetic reconstruction places *M. jacksoni* as sister to *Kallima* within nymphalid tribe Kallimini.

Jackson’s leaf butterfly, *Mallika jacksoni*, is a lepidopteran pollinator in banana–coffee plantations and is an indicator species of land-use change within tropical East African forests (Kühne et al. 2004; Munyuli 2012). *Mallika jacksoni* was originally classified in the Asian genus *Kallima* due to similarities in wing coloration related to leaf-mimicry masquerade camouflage (Skelhorn 2015), but all African ‘*Kallima*’ species were later reassigned based on significant structural differences in genitalia and behavior (Shirôzu and Nakanishi 1984). This species was assigned to the monotypic genus *Mallika* while the other African ‘*Kallima*’ species were either assigned to *Kamilla* (Larsen 1991) or to *Junonia* (Wahlberg et al. 2005).

Here, we report the complete mitochondrial genome sequence of *M. jacksoni* from specimen Mjack2011.1, collected at Mount Rom, Kitgum, Uganda (GPS 3.2783N, 32.8867E) in June 2011 that has been pinned, spread, and deposited in the Wallis Roughley Museum of Entomology, University of Manitoba (voucher WRME0507731).

DNA was prepared (McCullagh and Marcus 2015) and sequenced by Illumina NovaSeq6000 (San Diego, CA) (Marcus 2018). The mitogenome of *M. jacksoni* (GenBank MT704828) was assembled and annotated with reference to a *Junonia stygia* (Lepidoptera: Nymphalidae) mitogenome (MN623383) (Living Prairie Mitogenomics Consortium 2020) based on 8,850,239 paired 150 bp reads using Geneious 10.0.9 software. The *M. jacksoni* nuclear rRNA repeat (MT704831) was also assembled and annotated using a *J. stygia* (MN623382) reference sequence.

The *M. jacksoni* has a circular 15,193 bp mitogenome assembly composed of 13,009 paired reads with nucleotide composition: 39.1% A, 12.7% C, 8.0% G, and 40.3% T. The mitogenome gene order and composition is identical to all known butterfly mitogenomes (Cao et al. 2012; McCullagh and Marcus 2015). *Mallika jacksoni COX1* features an atypical CGA start codon as in many other insects (Liao et al. 2010). The mitogenome contains two protein-coding genes (*COX1*, *COX2*) with single-nucleotide (T) stop codons, and four protein-coding genes (*ATP6*, *ND3*, *ND4*, *ND5*) with two-nucleotide (TA) stop codons completed by post-transcriptional addition of 3′-A residues. The locations and structures of tRNAs were determined using ARWEN v.1.2 (Laslett and Canback 2008). tRNAs have typical cloverleaf secondary structures except trnS (AGN) where the dihydrouridine arm is replaced by a loop, while the mitochondrial rRNAs and control region are typical for Lepidoptera (McCullagh and Marcus 2015).

Mitogenomes from *M. jacksoni* and 39 additional specimens from tribes Junoniini, Kallimini, Nymphalini, and outgroup Melitaeini within subfamily Nymphalinae (McCullagh and Marcus 2015; Peters and Marcus 2017; Lalonde and Marcus 2019a, 2019b; Hamilton et al. 2020; Lalonde and Marcus 2020; Payment et al. 2020) were used for phylogenetic reconstruction. Sequences were aligned in CLUSTAL Omega (Sievers et al. 2011) and analyzed by parsimony and maximum likelihood (model selected by jModeltest 2.1.7) (Darriba et al. 2012) and likelihood ratio test (Huelsenbeck and Rannala 1997) in PAUP* 4.0b8/4.0d78 (Swofford 2002) (Figure 1). Considerable mitogenome divergence places *M. jacksoni* as a distinctive lineage within tribe Kallimini (sister to *Kallima*), as suggested previously on the basis of morphology (Larsen 1991) and molecular markers (Wahlberg et al. 2005).

**Figure 1.**
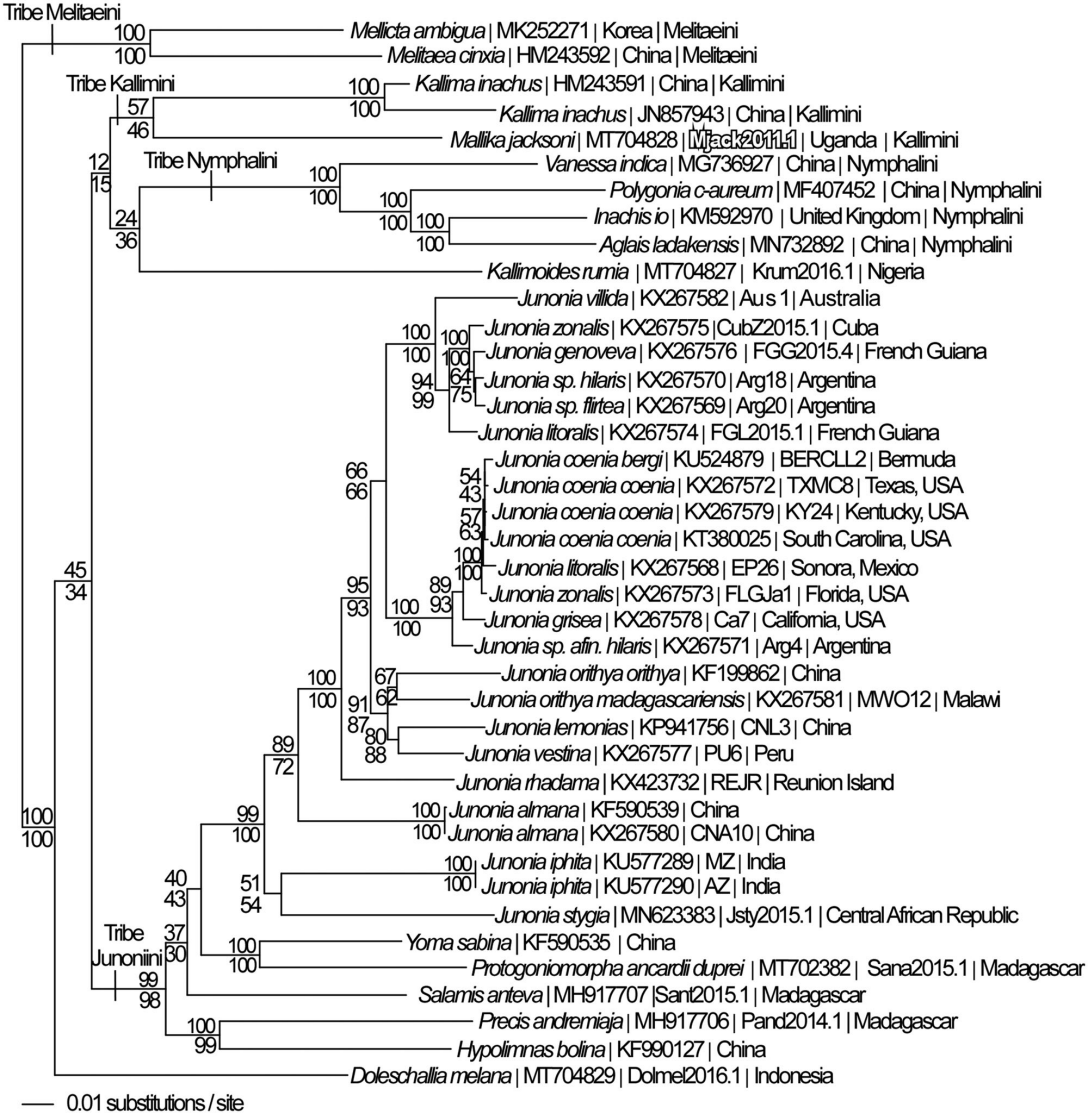
Maximum likelihood phylogeny (GTR + G model, G = 0.2250, likelihood score 109202.82577) of *Mallika jacksoni* and 39 additional mitogenomes from nymphalid subfamily Nymphalinae based on 1 million random addition heuristic search replicates (with tree bisection and reconnection). One million maximum parsimony heuristic search replicates produced eight trees (parsimony score 18,599 steps) which differ from one another only by the arrangement of *Junonia coenia* mitogenomes and one of which has an identical tree topology to the maximum likelihood tree depicted here. Numbers above each node are maximum likelihood bootstrap values and numbers below each node are maximum parsimony bootstrap values (each from 1 million random fast addition search replicates).

## Data Availability

The data that support the findings of this study are openly available in GenBank of NCBI at https://www.ncbi.nlm.nih.gov, reference numbers MT704828 and MT704831.
